# Genetic Characterization of Cupped Oyster Resources in Europe Using Informative Single Nucleotide Polymorphism (SNP) Panels

**DOI:** 10.3390/genes11040451

**Published:** 2020-04-21

**Authors:** Sylvie Lapègue, Serge Heurtebise, Florence Cornette, Erwan Guichoux, Pierre-Alexandre Gagnaire

**Affiliations:** 1 Ifremer, SG2M-LGPMM, 17390 La Tremblade, France; Serge.Heurtebise@ifremer.fr (S.H.); Florence.Cornette@ifremer.fr (F.C.); 2 BIOGECO, INRAE, University Bordeaux, F-33610 Cestas, France; erwan.guichoux@inrae.fr; 3 ISEM, Univ Montpellier, CNRS, EPHE, IRD, 34000 Montpellier, France; pierre-alexandre.gagnaire@umontpellier.fr

**Keywords:** cupped oysters, population genetics, introgression, conservation, shellfisheries

## Abstract

The Pacific oyster, *Crassostrea gigas*, was voluntarily introduced from Japan and British Columbia into Europe in the early 1970s, mainly to replace the Portuguese oyster, *Crassostrea angulata*, in the French shellfish industry, following a severe disease outbreak. Since then, the two species have been in contact in southern Europe and, therefore, have the potential to exchange genes. Recent evolutionary genomic works have provided empirical evidence that *C. gigas* and *C. angulata* exhibit partial reproductive isolation. Although hybridization occurs in nature, the rate of interspecific gene flow varies across the genome, resulting in highly heterogeneous genome divergence. Taking this biological property into account is important to characterize genetic ancestry and population structure in oysters. Here, we identified a subset of ancestry-informative makers from the most differentiated regions of the genome using existing genomic resources. We developed two different panels in order to (i) easily differentiate *C. gigas* and *C. angulata*, and (ii) describe the genetic diversity and structure of the cupped oyster with a particular focus on French Atlantic populations. Our results confirm high genetic homogeneity among Pacific cupped oyster populations in France and reveal several cases of introgressions between Portuguese and Japanese oysters in France and Portugal.

## 1. Introduction

Hybridization between species or evolutionary lineages is a common phenomenon in plant and animal species, including numerous animal groups, such as mammals, fish, birds, insects, and molluscs [1,2]. Such evidence of partial reproductive isolation has been of long-term interest for our understanding of speciation [3,4,5,6]. In the marine environment, the existence of such semi-isolated species pairs evolving in the “grey zone” of the speciation continuum [7] provides interesting opportunities to contribute to some highly debated questions in the field of speciation genomics. Because genetic divergence does not easily maintain in the face of gene flow in the absence of reproductive barriers [8], high gene flow species, such as broadcast-spawning marine invertebrates with highly dispersive pelagic larvae, offer valuable study systems for disentangling the mechanisms at play during speciation. For example, recent studies have revealed several cases of cryptic species subdivision even in broadcast-spawning marine invertebrates [9,10,11], confirming that semipermeable barriers to gene flow between closely related taxa [12] are relatively frequent in the marine realm.

Semi-isolated species pairs continue to evolve non-independently because some regions of their genome can still be exchanged through gene flow until speciation is complete. These exchanges of genetic material between divergent populations often occur during secondary contact episodes, as recently revealed by several population genomic studies [13,14,15,16]. Such scenarios are often met in the context of marine invasions and several studies have documented secondary gene flow between native and nonindigenous lineages [17,18]. In some cases, such as the green crab (*Carcinus maenas*), two closely related evolutionary lineages have been co-introduced and exchange genes though hybridization in the new invaded area [16].

Another original case comes from the Pacific cupped oyster, which has been taxonomically subdivided into two sister species, *Crassostrea gigas* and *C. angulata*. The two cryptic species are presumed to be parapatrically distributed in their native range in the Northwestern Pacific. *C. angulata* is mainly found in Southern China, Taiwan, and Vietnam [19], whereas *C. gigas* has a more northern distribution in northern China, Korea, Japan and Russia. The location of putative contact zones in the natural distribution range remains largely unknown. *C. angulata*, also called the Portuguese oyster, is presumed to have been non-voluntarily introduced in Portugal by merchant ships during the 16th century, probably from Taiwan [20,21], although the exact origins of introduced stocks remains unknown [22]. It was then introduced non-voluntarily in France by the end of the 19th century, and later for aquaculture purpose in other European countries, such as the United Kingdom, Belgium, the Netherlands, and Germany [23,24,25,26]. The Portuguese oyster replaced the native oyster species, the European flat oyster (*Ostrea edulis*), for shellfish farming, when this latter encountered overexploitation and disease problems during the 20th century. Recent studies have shown that *C. angulata* is currently widely distributed in Asian seas where it shows a high genetic diversity [19,27,28,29].

The Pacific oyster, *C. gigas*, was voluntarily introduced from Japan and British Columbia into Europe in the early 1970s, mainly to replace the Portuguese oyster in the French shellfish industry, following a severe disease outbreak [23]. Its introduction and settlement in Europe could be inferred using molecular markers [30]. Since then, the two species have been in contact in southern Europe (at least in the Ria Formosa lagoon) and, therefore, have the potential to exchange genes in a new environment compared to their native area [31,32]. At present, *C. angulata* has almost disappeared from Europe, being only found in Portugal in two rias, but also in the Cadiz region in Spain and Morocco [33]. The Pacific oyster *C. gigas* is now the species on which the European shellfish farming mostly relies on [34], but the species is also considered invasive, especially in Northern Europe.

Whether *C. gigas* and *C. angulata* truly represent biological species, semi-isolated species, or populations of the same species, has largely been discussed. The two taxa can be cross-fertilized in the laboratory to form viable and fertile offspring [35,36,37]. Based on this and their high genetic similarity, they have been considered as a single species [38,39]. However other studies reporting genetic differences between *C. angulata* and *C. gigas* supported their belonging to different but genetically closely related species [19,20,40,41]. This has been recently clarified by a genome-wide approach providing empirical evidence for highly heterogeneous levels of divergence across the genome, attributed to reduced introgression in low-recombining regions since secondary contact [42]. This finding implying the existence of partial reproductive isolation due to genetic barriers between the two cupped oysters shows that these semi-species are still evolving in the so-called “speciation grey zone”.

European shellfish farming is traditionally based on numerous transfers of animals of different stages within and among different bays from different countries. Hence, whatever the origin of the spats (i.e., natural beds or hatcheries), local populations of oysters likely result from complex histories of transport between different seashores. The growing of oysters from spat to adults in a single place is, rather, an exception. Therefore, as farmed oysters spawn in the sea, human-mediated transports of oysters for farming, may have resulted in complex spatiotemporal patterns of gene flow. As *C. angulata* still can be found in Southern Europe [33] and in places where *C. gigas* has been largely introduced for shellfish farming (Southern Portugal), recent genetic admixture is expected to happen. Natural hybridization has already been suggested in Portugal where the two species are in contact [31,32]. Moreover, *C. angulata* spat have been sold by a French hatchery since 2017, potentially increasing admixture opportunities in France or even in Europe. Furthermore, the two species display differences in resistance to diseases, growth, oxygen consumption, reproductive effort, time of settlement [32,43,44,45,46], but are difficult to distinguish on a morphological basis.

Thanks to the use of high throughput sequencing (HTS) approaches, thousands of polymorphic markers have been discovered [42,47], providing increased power to select subsets of ancestry informative loci to detect cryptic genetic subdivision and population structure [18]. For instance, a panel of 96 informative single-nucleotide polymorphisms (SNPs) allowed characterizing current ancestry patterns and temporal changes in admixture among two genetically distinct lineages of the invasive marine crab *Carcinus maenas*, which were independently introduced to eastern North America [48]. Here, we selected subsets of ancestry informative makers from existing genomic resources in order to (1) easily differentiate *C. gigas* and *C. angulata*, and (2) describe the genetic diversity and spatial structure of the cupped oyster with a particular focus on French Atlantic coasts. The newly developed panels revealed to be useful to characterize the cupped oyster resources present in Europe. We discuss our results in the context of a potential interest for conservation and management of cupped oyster resources.

## 2. Materials and Methods

### 2.1. SNP Panels

We genotyped four wild populations of *C. gigas* and *C. angulata* from both their native Asian and introduced Atlantic Ocean areas (see Biological Material section below) using two panels of SNPs. The first panel contained 384 SNPs developed in [49] and the second panel contained 10,144 Restriction-site Associate DNA sequencing (RAD-seq) derived SNPs from [42]. From each of the two published genotype datasets, we sorted SNPs according to their between-species *F_ST_* value. We chose unlinked SNPs with the highest *F_ST_* values to develop a panel of ancestry informative markers differentiating *C. gigas* and *C. angulata*. The characteristics of the 15 selected markers are given in Table S1.

A second panel was developed in order to further characterize within-species genetic structure among the cupped oyster populations. Therefore, we gathered 11 of the 15 ancestry informative SNPs from the previous panel with 69 other SNPs, chosen mainly from [49], but also [50]. The choice was performed according to their ability to produce reliable results, with regards to the description of genetic diversity, taking into account technical constraints associated with the development of two independent sets of 40 SNPs for genotyping on the MassARRAY system (Agena Bioscience, San Diego, CA, USA). These technical constraints explain why four SNPs from the 15 SNPs panel were not included. The characteristics of this second set of markers are given in Table S2.

### 2.2. Biological Material

#### 2.2.1. Biological Material Used to Develop the Ancestry Informative Panel (Dataset 1)

**Figure 1:**
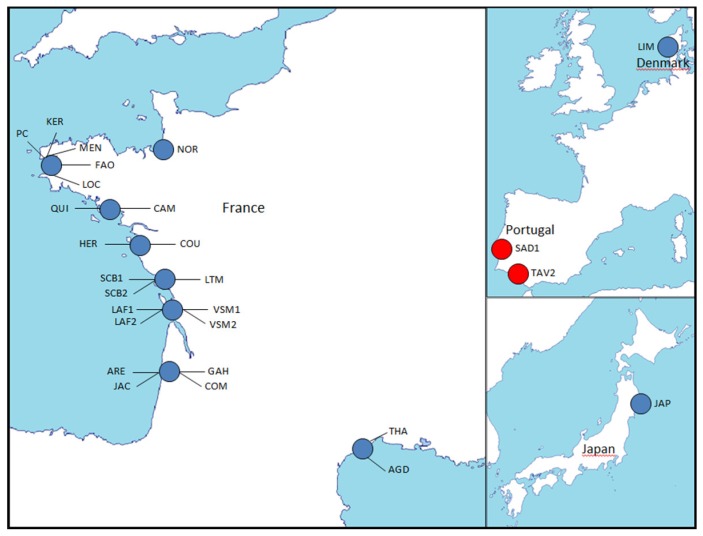
Location of the samples of the three datasets (see Table 1) used to characterize the cupped oyster diversity.

We sampled four wild populations of *C. gigas* and *C. angulata* from both their native Asian and introduced Atlantic Ocean areas. They are presented in Table 1 and Figure 1 and are the same populations as the ones used in [42], except that more individuals have been genotyped in the present dataset, and that some population name abbreviations have been modified here because of the sampling of the same populations at different times. Hence, the introduced French Marennes-Oléron Bay population (LAF) and Portuguese population (SAD) from [42] have been, respectively, renamed LAF1 and SAD1 in the present dataset. These two populations together with the native Japanese population (JAP) and the Taiwanese population (KEE) were used in the development of the 15 SNPs panel (Table 1). The genotypes of the 129 samples obtained with BeadXpress and/or RAD sequencing technology (see below) were used to calculate allelic frequencies, separately, in each population from each species at each of the 384 or 10 144 SNPs from [49] and [42], respectively.

#### 2.2.2. Biological Material Used to Confirm the Power of the Ancestry Informative Panel (Dataset 2)

In order to confirm the power of the 15 SNPs panel to differentiate *C. gigas* and *C. angulata* populations, we added four samples to the four previous populations (Table 1 and Figure 1). We obtained samples from another population from Portugal in the Algarve region (TAV1) as well as new alive animals from Marennes-Oléron Bay (LAFG0) and Sado River (SADG0). These were also used to experimentally produce a reference sample of F1 hybrids (HYB). Briefly, nine F1 biparental families were produced June 9, 2012, involving, respectively, five *C. gigas* females, five *C. angulata* males, four *C. angulata* females, and four *C. gigas* males (9 LFG0 and 9 SADG0). Between 11 and 12 animals of each family were sampled for this study for a total number of 104 HYB animals. Reproduction, larval, and post-larval rearing, as well as growing, took place at the Ifremer experimental hatchery in La Tremblade (Charente-Maritime, France). All of the 289 samples were genotyped thanks to one or two methods described below, mainly Sanger sequencing for the four added samples in this Dataset 2.

#### 2.2.3. Biological Material Used with the 80 SNPs Panel to Characterize the European Cupped Oyster Diversity (Dataset 3)

A large spatial scale sampling was performed in 2017 along the French Atlantic coast from Normandy to Arcachon Bay and along the Mediterranean Sea coast. It was completed by samples collected earlier (2010) in Brittany, Marennes-Oléron, and Arcachon Bay. The objective here was to characterize the genetic diversity of French cupped oyster populations across space and time, a decade after the beginning of important mortalities on spat, and more recently, adult oysters. Reference populations of *C. gigas* and *C. angulata* were added in this third study in order to characterize each species genetic background (*C. gigas* and *C. angulata*), as well as reference the North European population of *C. gigas* from Denmark. The 80 SNPs panel was used to genotype these 985 individuals. All of these populations are described in Table 1 and located (except for HYB) on Figure 1.

### 2.3. Genotyping Methods

DNA was extracted from gill tissues. A wizard^®^ DNA clean-up system (Promega, Madison, WI, USA) was used when the samples were collected in 70% or 90% alcohol, and a QiAamp DNA mini Kit (Qiagen, Hilden, Germany) when the samples where fresh or frozen. DNA quantification was performed with a Nanodrop spectrophotometer (Nanodrop Technologies, LLC, Wilmington, DE, USA).

According to the scale of the study and the number of SNPs used or expected, several genotyping methods were performed and combined: (1) genotyping by sequencing methods, such as Sanger sequencing and RADseq, and (2) genotyping methods with Illumina GoldenGate technology or MassARRAY system.

For the development of the 15 SNPs panel, the Illumina GoldenGate technology (Illumina Ins., San Diego, CA, USA) was used for part of the genotyping reactions, which were performed in accordance with the manufacturer’s protocol, as described in [49]. Briefly, data generated from the BeadXpress^®^ TM reader were analyzed with GenomeStudio for automated genotype clustering and calling. Clusters were then visually inspected to ensure high data quality. The RAD-derived SNP genotyping data were taken from [42].

A Sanger-based amplicon sequencing method was used for LAFG0, SADG0, HYB, and TAV1 samples. PCR primers were designed using the online Primer3 software package [51] based on the sequences available in [49] and [42]. Polymerase Chain Reaction (PCR) products were purified by alcohol precipitation using ammonium acetate. They were then sequenced in both directions using the ABI prism BigDye v3 Terminator Cycle sequencing Kit (Applied Biosystems, Foster City, USA) and the sequences were analyzed on an ABI 3130 XL genetic analyzer (Applied Biosystems) via Sequencing Analysis 5.2 to detect single-nucleotide polymorphisms.

SNP genotyping was performed for the 27 populations included in Dataset 3 using the iPLEX Gold chemistry following [52] on a MassARRAY System (Agena Bioscience). Data analysis was done using MassARRAY Typer Analyzer 4.0.26.75 (Agena Biosciences). We excluded monomorphic SNPs, loci with weak or ambiguous signal and loci high missing data.

As described above, the samples were genotyped by several methods, with often some common samples between the methods in order to confirm the identity of the genotypes. In those cases, no discrepancies were observed between the results obtained by the different methods. The genotypes of the three datasets are available at https://doi.org/10.17882/71545 [53].

### 2.4. Population Genetics Analyses

Genetic differentiation between pairs of populations was estimated SNP by SNP using *F_ST_* [54] in Genetix 4.1 [55]. Within- and between-population components of genetic diversity were decomposed using a discriminant analysis of principal components (dAPC), in order to maximize genetic variation between populations while minimizing within-population variation. The dAPC analysis was performed with the R package Adegenet [56].

Bayesian clustering of the genetic data was performed using Structure version 2.1 [57,58]. We ran Structure with K varying from 1 to 10, with 10 runs for each K value, to find the K value with the highest posterior probabilities, and used the ΔK statistics to evaluate the change in likelihood [59]. Our parameters were 10,000 burn-in periods and 50,000 Markov chain Monte Carlo repetitions after burn-in. For the most likely number of clusters, we calculated the average result over 10 runs to get the final admixture analysis.

## 3. Results

### 3.1. Development of Ancestry Informative SNP Panels

**Figure 2:**
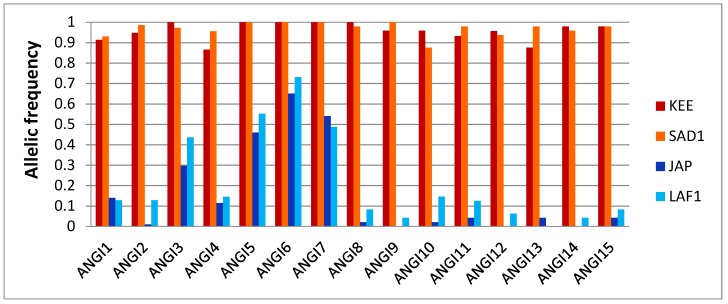
Allelic frequencies of the 15 single-nucleotide polymorphisms (SNPs) at the four native and introduced populations of *C. gigas* (in blue) and *C. angulata* (in red).

Using four populations from both species (two native and two introduced), we calculated allele frequencies within each population and estimated *F_ST_* between pairs of populations at each SNP. Although we did not find any fixed difference in allele frequency between the two species using the four populations sampled, we sorted all the SNPs according to the *F_ST_* between pairs of interspecific populations and chose the seven and eight SNPs with the higher values respectively in the BeadXpress (384) and RAD sequencing (10,144) panels. The frequency of the most frequent allele in *C. angulata* (named the “angulata” allele) at each SNPs in each of the four populations is presented in Figure 2. SNPs named ANGI1 to ANGI7 come from the BeadXpress panel [49] and SNPs named ANGI8 to ANGI15 were developed from the RAD sequencing panel [42]. The markers characteristics are detailed in Table S1. As illustrated in Figure 2, although the “angulata’ allele was sometimes fixed in one or even the two populations of *C. angulata* (markers ANGI3, 5, 6, 7, 8, and 9), it was always present in at least one of the two *C. gigas* populations (e.g., ANGI9). Reciprocally, “gigas” alleles that were fixed in one population of *C. gigas* (markers ANGI9, 12, 13, and 14) were found segregating in *C. angulata*. Therefore, none of these 15 markers, selected among the most highly differentiated regions of the genome, was fully diagnostic. We also found differences in the extent of allele frequency differences between *C. angulata* and *C. gigas* populations according to the fact that the SNP was extracted from the BeadXpress panel (ANGI1 to 7) or the RAD sequencing panel (ANGI8 to 15). This reflected a stronger effort to detect the most strongly differentiated markers in the RAD dataset compared to the BeadXpress SNP dataset.

### 3.2. Ancestry Informative Panels Differentiate Crassostrea gigas and Crassostrea angulata

**Figure 3:**
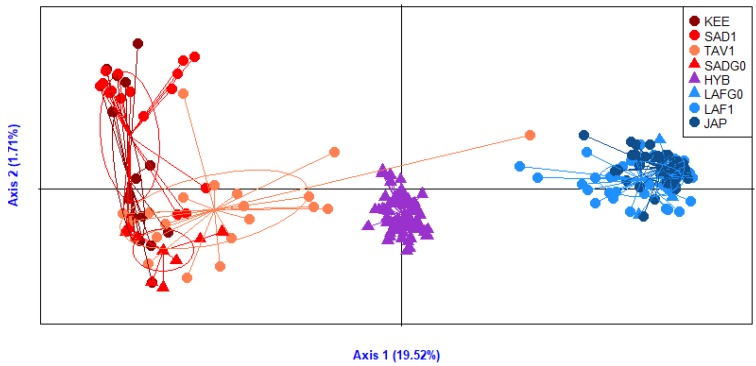
Discriminant analysis of principal components (dAPC) representation of the eight populations/progeny of cupped oysters, with *C. angulata* from Asia (KEE, dark red) and Europe (SAD1, TAV1, and SADG0, red), *C. gigas* from Asia (JAP, dark blue) and Europe (LAF1, LAFG0, blue), and experimental hybrids (HYB, violin). Natural populations (KEE, SAD1, TAV1, LAF1, JAP) are presented with a dot whereas G0 parents (SADG0, LAFG0) and experimental hybrids (HYB) are presented with a triangle.

The between-population genetic structure revealed by the dAPC (Figure 3) separated well the two species along the first axis (explaining 19.52% of total variance). As expected, the F1 hybrids produced in experimental conditions appeared in the middle of the first axis in between *C. gigas* and *C. angulata* samples. Although not a single SNP was differentially fixed between species, the combination of 15 SNPs with high *F_ST_* values in interspecific population pairs was able to clearly distinguish the populations from the two species and their hybrids. The second axis principally separated *C. angulata* samples with 1.71% of the total variance explained. Moreover, the PCA indicated potential events of introduction/introgressions in natural populations. For example, one TAV1 sample was found in the *C. gigas* part of the DAPC and four TAV1 samples appeared close to the hybrid group. More generally, the TAV1 population was slightly shifted toward the center of the first axis, potentially indicating increased introgression from *C. gigas* in this population closely located to the contact zone.

### 3.3. Diversity and Differentiation of the Cupped Oyster in France

**Figure 4:**
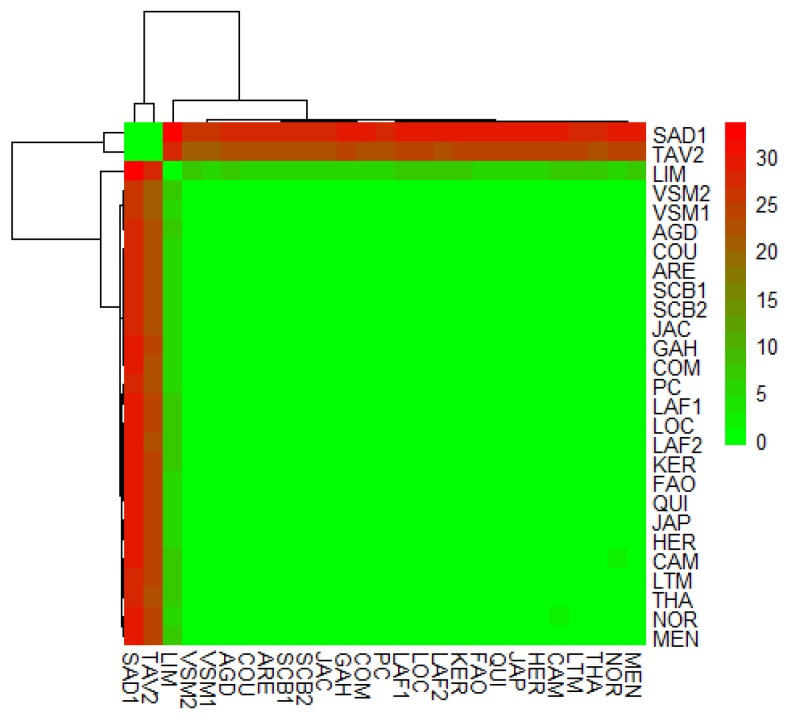
Pairwise *F_ST_* matrix (displayed in percentage) in the large scale Dataset 3.

The genetic diversity within populations of the large-scale study was highly homogenous in French and the Japanese populations, whereas it was lower in Norway and Portugal (Figure S1). Regarding the genetic structure of the European cupped oyster populations sampled, we found a global homogeneity at both the spatial and temporal scales. Similarly, all pairwise *F_ST_* estimates (Figure 4) were not significantly different from zero, except the pairwise values with LIM (Denmark) and with the two Portuguese samples, SAD1 and TAV2. Hence, all the French samples (from the Atlantic Ocean and Mediterranean Sea) and the Japanese reference could not be differentiated. Furthermore, there was no temporal differentiation between samples collected in 2010 and 2017 in three different bays. Significantly, *F_ST_* values varied from 0.045 to 0.069 between LIM and the French and Japanese populations, and from 0.21 to 0.334 between the *C. gigas* and *C. angulata* oyster populations (SAD1, TAV2).

**Figure 5:**
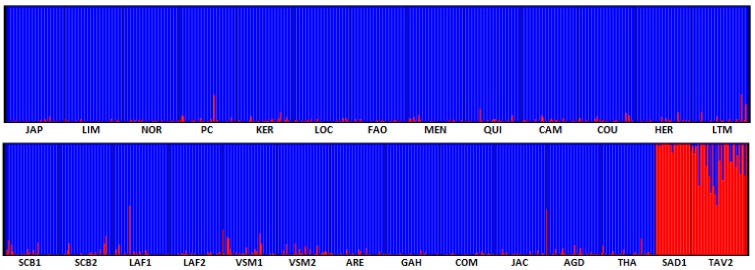
Individual Bayesian assignment proportion for K = 2 clusters. The abbreviation of the populations are given in Table 1. Blue and red colors correspond to the *C. gigas* and *C. angulata* genetic backgrounds respectively.

This global pattern was confirmed by the assignment of individuals to two genetic clusters (Figure 5), which correspond to the *C. gigas* and *C. angulata* genetic background (respectively, in blue and red). The two *C. angulata* populations SAD1 and TAV2, however, showed a different pattern. SAD1 was considered as a non-admixed reference population for *C. angulata* in Europe. By contrast, TAV2 showed a high number of admixed individuals. This result is confirmed by the position of TAV1 (although from a different sampling year) in the dAPC analysis (Figure 3). Furthermore, the *F_ST_* estimates between SAD1 and the other *C. gigas* populations varied from 25.7% to 33.4%, whereas the *F_ST_* estimates between TAV2 and the other *C. gigas* populations only varied from 21% to 26.8%. Our results also highlighted lower proportions of introgression in the French *C. gigas* populations. We indeed observed a high number of slightly introgressed samples in some French populations. This was especially true for populations VSM1 and VSM2 and to a lesser extent for populations SCB1 and SCB2. Furthermore, we also observed few highly introgressed individuals in several populations. This was particularly the case for one individual in LAF1 and one individual in JAC. However, when looking at the 90% confidence intervals of the ancestry proportions of each individual, to each of the two clusters inferred with structure, we observed that only one individual from LAF1 and one individual from JAC showed an assignment probability to each cluster significantly different from 0 or 1 (compared to 9 individuals from TAV2).

## 4. Discussion

### 4.1. A New Flexible Tool to Differentiate the Two Oyster Resources in Europe

Although HTS has allowed the development of medium-density SNP panelsand whole genome resequencing of hundreds of individuals in oysters [47,60,61,62], those approaches do not always scale to specific questions that only require small SNP panels. The level of precision for inferring individual ancestry for species identification requires only a handful of ancestry informative markers, typically one or two per chromosome. These can be identified using traditional genome scans for “outlier” loci, in order to select markers showing the strongest levels of genetic differentiation between groups of populations belonging to different species. This is what we did here to distinguish the two closely related species *C. gigas* and *C. angulata*. On the basis of two previously published sets of SNP markers [42,49], we selected SNPs showing the highest *F_ST_* values between pairs of interspecific populations. Although our final panel consisted of 15 SNPs that were evenly chosen from the two initial datasets, the most informative markers were mainly obtained from the RADseq SNPs panel (e.g., ANGI9, 10, 14, and 15). This simply reflects the fact that this high-dimensional SNP dataset offered a far higher number of SNPs, among which the most significant outlier loci could be searched. This was also illustrated in Figure 2, in which the SNPs from the RADseq SNPs panel (e.g., ANGI8 to 15) showed more extreme allele frequency differences between *C. gigas* and *C. angulata* populations. The overall same amount of variance was explained by axis 1 (19.52%) on the discriminant analysis of Dataset 2 than in [42]. Since we used an extended sampling to evaluate the discrimination power of these SNPs, this result confirms that the 15 “outlier” SNPs used in this panel are sufficient to evaluate individual ancestry with a reasonable precision.

This new panel of 15 SNPs is clearly more powerful than the few markers already available [31,32] even though none of these SNPs individually appeared to be fully diagnostic. This reflects the fact that the genomic landscape of divergence between *C. gigas* and *C. angulata* does not contain any region with fixed differences, as previously observed using more than 10 thousand SNPs [42]. In this context, the interest of the tool developed here lies in its capacity to quantify mixed ancestries between the two species with a reasonable degree of precision and a very low cost. Hence, with 15 ancestry-informative SNPs (Dataset 2) or 11 of these 15 SNPs included in a larger panel of 80 SNPs (Dataset 3), it was possible to identify several cases of admixed ancestry. This should be of high interest for experimental and commercial hatcheries working with both species, enabling them to follow the diversity and levels of introgression in their broodstock.

### 4.2. Different Cases of Introgression

A more challenging aspect of using small SNP panels to characterize individual ancestry in oysters is the detection of introgression. Shared variation between the two species across their genome has been attributed to postglacial gene flow in addition to incomplete lineage sorting (ILS) [42]. Although our SNP panels are efficient to detect first generation hybrids and admixed genotypes with balanced ancestry proportions, the detection of introgressed individuals that are mostly made of a given species ancestry might be more problematic. In order to evaluate our capacity to detect recent introgression, we compared ancestry patterns between pairs of populations from close geographic locations. In the presence of ILS and historical gene flow alone, such samples should exhibit similar ancestry patterns.

In Portugal, such a comparison allowed us to evidence introgression within *C. angulata*. Our samples from the Sado estuary (samples SADG0 and SAD1), which represents one of the last sites where the Portuguese oyster is known to be present in Europe [31,33], and where *C. gigas* is presumed to be absent or rare [22], were inferred as non-admixed *C. angulata* ancestries. By contrast, the Ria Formosa lagoon (samples TAV1 and TAV2) displayed a completely different situation with a high rate of introgression from *C. gigas*, which is known to have been introduced for more than 15 years for aquaculture purpose [31]. In this sample, ancestry proportions varied among individuals, consistent with recent introgression. Although an asynchrony in settlement time has been characterized in this lagoon [32], it does not appear important enough to impede the ongoing introgression.

In France, the studied populations of *C. gigas* also displayed varying rates of *C. angulata* ancestry. Sampling locations such as VSM1 and 2, or SCB1 or 2, contained a high number of slightly introgressed animals compared to all other locations. This suggests that recent gene flow has been relatively more pronounced in these locations. This situation might be a remainder of the ancient presence of *C. angulata* 50 years ago. Hence before *C. angulata* almost disappeared and *C. gigas* invaded France, both species could have been in contact during several years [23]. This may particularly have happened in most isolated areas, such as at the extreme west of Ré Island (SCB samples) or in the Gironde estuary (VSM samples). In this latter area, a different behavior of the oysters has been observed by local farmers (Regional Marennes-Oléron group of shellfish farmers, personal communication). Moreover, the two sampling locations, VSM1 and SCB1, had already shown a very slight but significant differentiation with other French and Japanese sites that was attributed to their isolated situation [63]. This could represent an inverted picture of what possibly happened in Ria Formosa lagoon within the last decades. However, those results were not statistically supported, certainly because of a low number of markers and a lack of statistical power, and need to be confirmed. We also detected a few significantly highly introgressed individuals in Marennes-Oléron bay (LAF1) or Arcachon bay (JAC). These genotypes could represent recent generation hybrids reflecting ongoing gene flow in these locations. This could be favored by numerous exchanges between French and Portuguese breeding sites and the potential release of *C. angulata* hatchery spat. This alternative explanation is, however, less likely to involve a fully inverted picture as the one evidenced in Portugal, due to the very high number of *C. gigas* bred in France.

Quantifying gene flow between *C. angulata* and *C. gigas* and its evolutionary consequence is clearly needed, at least in Portugal and France. Therefore, samples collected regularly in successive years should be analyzed, possibly with a higher number of markers to increase statistical power, and beginning with the populations already identified as introgressed, whatever the reasons underlying this situation.

### 4.3. Conservation Issues

The detection of hybridization also leads to conservation issues and even policy implications as it is related to the integrity of the genetic structure of populations [64]. Being able to identify introgressed populations, and even the presence of different hybrid classes [65], is particularly challenging when the degree of genetic differentiation among groups is low, but it is necessary to attempt to address conservation issues. This is particularly true for oysters for with the level of genetic relatedness is important but for which, however, differences in phenotypic traits potentially of interest can be observed such as resistance to diseases, growth, physiological behavior, especially in a rapid changing environment.

Conservation issues are especially relevant to the preservation of the least introgressed populations of *C. angulata*. This could be seen as a paradox as both species are introduced species, with *C. gigas* being even considered in some places as invasive and noxious [66,67,68]. However, they are also considered as naturalized species in southern Europe where shellfish farming has relied on for about 100 years. It is even more the case for the Portuguese oyster, the aptly named species, which is considered as a patrimonial species in Portugal. Besides, the Scandinavian spread of *C. gigas* is well documented [26,69], showing that despite high winter [70] and summer mortalities [71], the species has increased in densities especially since 2007 and is now well established in Scandinavian waters [72]. Aquaculture experiments were at the origin of such an establishment and it is interesting to notice that in Norway, even after import regulations became stricter [73], and the retraction of the last cultivation licenses, the spread could not be stopped. There are few published data on eradication or reduction programs targeted at *C. gigas*, but for example, from Australia [68], where *C. gigas* is considered a noxious species in areas where it can outcompete native oysters. The Netherlands is the only European country where significant mass reduction trials have been conducted [67]. This illustrates the difficulty to mitigate a range extension when launched especially for an invasive species such as *C. gigas*. Furthermore, transnational management might be hard to achieve especially when there are variations between countries, regions, or even between stakeholders on the perception of a species to be a menace or a resource. In this context managing the spread of an invasive species even at early stages of expansion [74] might be too late in the Ria Formosa lagoon case.

Therefore, conservation and/or restoration actions in specific Portuguese rias could be considered for the Portuguese oyster. As being considered a naturalized species in Europe, and almost an indigenous one in Portugal, *C. angulata* conservation and/or restoration actions could be inspired by some programs launched for real indigenous oysters. Interesting examples can be found in Northern Europe concerning the European native flat oyster, *Ostrea edulis*. After having suffered from overexploitation and diseases during the twentieth century, those oyster reefs are now seen, far beyond a valuable food resources, as a characteristic benthic community, which offers the possibility of biodiversity enhancement and ecosystem services in the marine environment [75,76]. Currently, restoration projects are carried out in England, Scotland, Ireland, France, the Netherlands, Germany, and Sweden. The Native Oyster Restoration Alliance (NORA) recently settled network should help those national projects to be extended at the European level [77]. An encouraging restoration example of the American cupped oyster, *Crassostrea virginica*, took place during the last 10 years [78] and showed that beyond a quantitative success it was possible to very rapidly reach an equivalent level of genetic diversity in the restored reefs as in the undisturbed ones.

Concerning the *C. gigas* and *C. angulata* species, and besides ecosystem services, conserving one endangered resource (*C. angulata*) is of particular interest as they also differ by several phenotypic traits. Such a different resource, if less economically interesting at present, might become more when considering the rapid evolution of the marine environment. However more studies are needed in Europe to be able to exclude phenotypic plasticity as a source of quantitative differences, which requires careful common-garden studies [79], such as performed in China where divergent adaptive strategies were observed with underlying evolutionary trade-offs between genetic adaptation and plasticity at the molecular level in the two oyster congeners [47,80,81].

### 4.4. Confirmation of a High Homogeneity within the Pacific Cupped Oyster Populations in France

In non-native species, we may expect founder effects [82] on the basis of the introduction of a small number of individuals to preclude the success of introductions. However including multiple and repeated introductions, as well as individual introductions with extremely high propagule loads, increases the probability to reduce this founder effect, to introduce “preadapted” genotypes, and/or to provide sufficient diversity on which selection may act [83]. This might be the case for the Pacific oyster *C. gigas*, for which several repeated introductions from Canada and Japan of adults, but also high quantities of spat, happened by the end of the 1960s in Europe, especially in France and the Netherlands [23,84]. Hence our results confirm an absence of detection of clear differences between the source population and the French populations of *C. gigas* as several other studies did even with a far higher number of markers [21,42,50,61,85,86]. We failed at detecting differences between Asian and European populations within each species [42] because our 80 SNPs panel was not well adapted for detecting such structure if it exists. However this panel allowed detecting the difference observed between the southern group of European *C. gigas* populations and the northern group [21,30,42,50,61,86,87] here represented by the Danish sample (LIM). Hence several outlier loci between the two groups identified in [50] had been included in this panel. Thus, this panel answers two objectives: a powerful differentiation between *C. gigas* and *C. angulata* and the differentiation between northern and southern European populations. Concerning the French populations sampling, we also confirm a high genetic homogeneity as already observed with several microsatellite markers [63,88] with the exception of SCB and VSM samples already mentioned in 2011 that still need to be confirmed. The combination of the biological traits of the species and the farmer’s practices of translocation compete to a very important gene flow within and between wild and farmed populations. No loss of diversity or even differentiation was observed between the same sites sampled in 2010 and 2017. The hypothesis tested here was the potential impact of massive mortalities that has happened on spat since 2008, and adults since 2012, on the genetic structure of those populations. This result may indicate that such an impact was negligible or at least at the time scale of our sampling. However, a bias regarding this observed spatial or temporal genetic homogeneity may come from the low-density panel that would have been unable to detect a weak difference. Our results, however, confirm those of [50], obtained with several hundreds of markers. The more recent studies using tens of thousands of markers did not include several French wild samples in their studies [42,61]. However, including several of our French samples in a genome-wide analysis together with introgressed *C. gigas*/*C. angulata* Portuguese and French samples could be of interest, as a recent study on Chinese *C. gigas* populations, performed with the whole-genome re-sequencing of 371 oysters, provided evidence of a weak but significant differentiation among oyster populations at fine spatial scales [47].

## Figures and Tables

**Table 1:** Characteristics of the locations sampled.

Population Abbreviation	Location	Origin	Country	GPS Coordinates	Sampling (Production) Year	Sample Size	Dataset
KEE	Keelung	Keelung bay	Taïwan	25°09’29.89"N - 121°44’54.87"E	1996	28	1.2
SAD1	Rio Sado	Sado estuary	Portugal	38°29’15.72"N - 8°49’44.48"O	2010	36	1,2,3
JAP	Miyagi	Matsushima bay	Japan	38°21’31.99"N - 141°6’20.34"E	2010	47	1,2,3
LAF1	Pointe de La Fumée	Charente estuary	France	46°00’10.01"N - 1°07’17.46"O	2010	39	1,2,3
LAFG0	Pointe de La Fumée	Charente estuary	France	46°00’10.01"N - 1°07’17.46"O	2012	9	2
SADG0	Rio Sado	Sado estuary	Portugal	38°29’15.72"N - 8°49’44.48"O	2012	9	2
HYB	Experimental hatchery	LAFG0 X SADG0	France		(2012)	104	2
TAV1	Tavira	Algarve	Portugal	37°06’59.98"N - 7°37’41.21"O	1998	24	2
LIM	Limfjorden	Jutland	Denmark	56°43’16.99"N - 8°15’26.34"E	2010	38	3
NOR	Champeaux	Mont Saint-Michel bay	France	48°43’50.80"N - 1°31’59.76"O	2010	37	3
PC	Pointe du Château	Brest bay	France	48°19’51.44"N - 4°18’60.00"O	2010	37	3
KER	Kersanton	Brest bay	France	48°21’N - 4°17’O	2010	37	3
LOC	Le Loc’h - Anse de Poulmic	Brest bay	France	48°17’32.40"N - 4°20’11.20"O	2017	37	3
FAO	Faou river	Brest bay	France	48°17’51.70"N - 4°13’66.30"O	2017	37	3
MEN	Daoulas river - Mengleuz	Brest bay	France	48°20’62.80"N - 4°17’37.10"O	2017	37	3
QUI	Kerivor	Quiberon bay	France	47°34’57.40’’N - 3°06’41.80"O	2017	37	3
CAM	Camaret	Vilaine bay	France	47°29’79.40"N - 2°29’46.50"O	2017	37	3
COU	La Couplasse	Bourgneuf bay	France	47°00’45.78"N - 2°01’02.78"O	2017	37	3
HER	L’Herbaudière	Noirmoutier island	France	47°01’13.72"N - 2°18’15.60"O	2017	37	3
LTM	La Tranche sur mer	Aiguillon bay	France	46°20’0.10"N - 1°21’0.60"O	2010	37	3
SCB1	Saint-Clément des Baleines	Ré island	France	46°14’43.62"N - 1°33’47.56"O	2010	37	3
SCB2	Saint-Clément des Baleines	Ré island	France	46°14’43.62"N - 1°33’47.56"O	2017	36	3
LAF2	Pointe de La Fumée	Charente estuary	France	46°00’10.01"N - 1°07’17.46"O	2017	36	3
VSM1	Vaux sur mer	Gironde estuary	France	45°37’51.13"N - 1°03’53.46"O	2010	38	3
VSM2	Vaux sur mer	Gironde estuary	France	45°37’51.13"N - 1°03’53.46"O	2017	36	3
ARE	Arès	Arcachon bay	France	44°40’13.55’’N - 1°04’35.45"O	2010	37	3
GAH	Gahignon	Arcachon bay	France	44°42’26.93"N - 1°8’11.55"O	2010	37	3
COM	Comprian	Arcachon bay	France	44°40’13.55"N - 1°04’35.45"O	2017	37	3
JAC	Jacquets	Arcachon bay	France	44°72’18.50"N - 1°18’79.50"O	2017	36	3
AGD	Cap d’Agde	Mediterranean sea	France	43°16’44.48"N - 3°29’45.13"E	2010	36	3
THA	Thau lagoon	Mediterranean sea	France	43°26’80.00"N - 3°39’47.70"E	2017	37	3
TAV2	Tavira	Algarve	Portugal	37°06’59.98"N - 7°37’41.21"O	2000	38	3

* Reference to the dataset the samples where used in: 1 = development of the ancestry informative panel; 2 = confirmation of the power of the ancestry informative panel; 3 = characterization of the European cupped oyster diversity.

## Supplementary Materials

The following are available online at https://www.mdpi.com/2073-4425/11/4/451/s1. Figure S1: Estimated and observed heterozygosity in the large-scale population Dataset 3. Table S1: List of the ancestry informative SNP panel. Table S2: List of the 80 SNPs used to characterize the cupped oyster genetic diversity.
